# Characteristics and outcomes of elderly patients with diffuse gliomas: a multi-institutional cohort study by Kansai Molecular Diagnosis Network for CNS Tumors

**DOI:** 10.1007/s11060-018-2957-7

**Published:** 2018-08-03

**Authors:** Takahiro Sasaki, Junya Fukai, Yoshinori Kodama, Takanori Hirose, Yoshiko Okita, Shusuke Moriuchi, Masahiro Nonaka, Naohiro Tsuyuguchi, Yuzo Terakawa, Takehiro Uda, Yusuke Tomogane, Manabu Kinoshita, Namiko Nishida, Shuichi Izumoto, Yoshikazu Nakajima, Hideyuki Arita, Kenichi Ishibashi, Tomoko Shofuda, Daisuke Kanematsu, Ema Yoshioka, Masayuki Mano, Koji Fujita, Yuji Uematsu, Naoyuki Nakao, Kanji Mori, Yonehiro Kanemura

**Affiliations:** 1 Department of Neurological Surgery, Wakayama Medical University School of Medicine, Kimiidera 811-1, Wakayama, 641-0012 Japan; 2 Kansai Molecular Diagnosis Network for CNS Tumors, Osaka, Japan; 3 Department of Pathology and Applied Neurobiology, Graduate School of Medical Science, Kyoto Prefectural University of Medicine, Kyoto, Japan; 4 Department of Diagnostic Pathology, Hyogo Cancer Center, Hyogo, Japan; 5 Department of Neurosurgery, Osaka National Hospital, National Hospital Organization, Osaka, Japan; 6 Department of Neurosurgery, Rinku General Medical Center, Izumisano, Osaka Japan; 7 Department of Neurosurgery, Kansai Medical University, Osaka, Japan; 8 Department of Neurosurgery, Osaka City University Graduate School of Medicine, Osaka, Japan; 9 Department of Neurosurgery, Hyogo College of Medicine, Nishinomiya, Hyogo Japan; 10 Department of Neurosurgery, Osaka International Cancer Institute, Osaka, Japan; 11 Department of Neurosurgery, Tazuke Kofukai Foundation, Medical Research Institute, Kitano Hospital, Osaka, Japan; 12 Department of Neurosurgery, Kindai University Faculty of Medicine, Osaka, Japan; 13 Department of Neurosurgery, Sakai City Medical Center, Osaka, Japan; 14 Department of Neurosurgery, Osaka University Graduate School of Medicine, Osaka, Japan; 15 Department of Neurosurgery, Osaka City General Hospital, Osaka, Japan; 16 Division of Stem Cell Research, Department of Biomedical Research and Innovation, Institute for Clinical Research, Osaka National Hospital, National Hospital Organization, Osaka, Japan; 17 Division of Regenerative Medicine, Department of Biomedical Research and Innovation, Institute for Clinical Research, Osaka National Hospital, National Hospital Organization, Osaka, Japan; 18 Department of Pathology, Osaka National Hospital, National Hospital Organization, Osaka, Japan; 19 Department of Neurosurgery, Kansai Rosai Hospital, Hyogo, Japan

**Keywords:** Elderly, Diffuse glioma, Glioblastoma, Molecular marker, Prognostic factor, Real-world data

## Abstract

**Introduction:**

This study investigates the current state of clinical practice and molecular analysis for elderly patients with diffuse gliomas and aims to elucidate treatment outcomes and prognostic factors of patients with glioblastomas.

**Methods:**

We collected elderly cases (≥ 70 years) diagnosed with primary diffuse gliomas and enrolled in Kansai Molecular Diagnosis Network for CNS Tumors. Clinical and pathological characteristics were analyzed retrospectively. Various factors were evaluated in univariate and multivariate models to examine their effects on overall survival.

**Results:**

Included in the study were 140 elderly patients (WHO grade II: 7, III: 19, IV: 114), median age was 75 years. Sixty-seven patients (47.9%) had preoperative Karnofsky Performance Status score of ≥ 80. All patients underwent resection (gross-total: 20.0%, subtotal: 14.3%, partial: 39.3%, biopsy: 26.4%). Ninety-six of the patients (68.6%) received adjuvant treatment consisting of radiotherapy (RT) with temozolomide (TMZ). Seventy-eight of the patients (75.0%) received radiation dose of ≥ 50 Gy. *MGMT* promoter was methylated in 68 tumors (48.6%), *IDH1*/*2* was wild-type in 129 tumors (92.1%), and *TERT* promoter was mutated in 78 of 128 tumors (60.9%). Median progression-free and overall survival of grade IV cases was 8.2 and 13.6 months, respectively. Higher age (≥ 80 years) and *TERT* promoter mutated were associated with shorter survival. Resection and adjuvant RT + TMZ were identified as independent factors for good prognosis.

**Conclusions:**

This community-based study reveals characteristics and outcomes of elderly glioma patients in a real-world setting. Elderly patients have several potential factors for poor prognosis, but resection followed by RT + TMZ could lengthen duration of survival.

**Electronic supplementary material:**

The online version of this article (10.1007/s11060-018-2957-7) contains supplementary material, which is available to authorized users.

## Introduction

Diffuse gliomas are the most common primary central nervous system tumors, accounting for about 30% of all brain tumors in Japan [1]. Median age at glioblastoma (WHO grade IV) diagnosis is 63.0 years and higher than median age at diagnosis of lower-grade gliomas [1]. Recently, the number and percentage of elderly people are rising in Japan, and hence diffuse gliomas in the elderly are becoming more common [1, 2]. As this demographic tends to fare worse than non-elderly population, there are major concerns regarding prediction of clinical behavior and treatment outcomes.

In clinical practice, physicians are usually apprehensive in offering aggressive treatments to elderly patients because of concerns relating to treatment tolerance due to advanced age, co-morbidities or underlying propensity for complications [3, 4]. Although treatment-associated toxicity in the elderly appears to be higher and optimal treatment for elderly patients remains controversial, treatment tolerance seems to be dependent on individual predisposition as well as comorbid conditions [5–8]. Clinical consequences are often complicated by additional considerations common to elderly populations.

Adult diffuse gliomas have highly variable clinical behavior, response to therapy, and outcomes [9, 10]. Recently, mutations in *IDH, TP53, TERT* promoter and codeletion of chromosome arms 1p and 19q (1p/19q codeletion) have been highlighted as clinically relevant prognostic markers of diffuse gliomas [9–13]. Some of these molecular parameters are required for integrative diagnosis for 2016 CNS WHO Classification [14]. Some molecular markers have also been reported as being predictive of the potential benefit from specific therapeutic intervention. Particularly in elderly patients, *MGMT* promoter methylation status is reportedly to be important information for deciding adjuvant treatment regimen [8, 15–17]. The prevalence and impact of previously established biomarkers are considered as a main area of investigation for diffuse gliomas in the elderly.

This study aims to demonstrate the current state of clinical practice for elderly patients with diffuse gliomas and molecular analyses of diffuse gliomas in the elderly. In the multi-institutional retrospective cohort study of 140 elderly cases treated at 13 hospitals in Kansai Molecular Diagnosis Network for CNS Tumors (Kansai Network), we elucidate both clinical and pathological features of elderly glioma cases, as well as treatment outcomes and prognostic factors of glioblastoma (GB) patients in a real-world setting.

## Methods

### Ethics

This study was carried out in accordance with the principles of the Helsinki declaration. Approval was obtained from the Institutional Review Board of Wakayama Medical University (No. 98), Osaka National Hospital (No. 713), and all collaborative institutes. Written informed consent was obtained from all patients.

### Patient population

This study included patients (≥ 70 years) who were treated at 13 institutions or hospitals participating in the Kansai Network. Established in the Kansai area of Western Japan, the Kansai Network collects tumor samples and clinical information from affiliated hospitals and analyzes molecular status of tumors for diagnosis and research. Between September 2007 and September 2016, we collected total 918 samples including all kinds of primary and recurrent gliomas. From this data bank, we focused on primary diffuse gliomas of the elderly and collected elderly cases (Online Resource 1). Each institution provided between two and 20 patients. Diagnosis of diffuse gliomas was initially confirmed by histopathological examination at each institution or hospital (Online Resource 1).

### Clinical information

Clinical information was collected from medical records including patient demographics, preoperative KPS scores, extent of surgical resection (EOR), adjuvant radiation and chemotherapy (RCT) regimens, and survival time. EOR was classified as gross total resection (GTR, > 95% of the tumor was resected), subtotal resection (STR, 90–94%), partial resection (PR, < 90%) and biopsy according to the assessment by the surgeon. Patients received RCT consisting of radiation (RT) plus concomitant and adjuvant temozolomide (TMZ), RT alone, TMZ monotherapy or none [18]. Adjuvant RCT regimens were determined by attending physicians considering the patient’s condition.

### Histopathological examination

All cases underwent central pathology review by senior board-certified neuropathologists (Y.K. and T.H.). Integrated diagnosis and WHO grading were made based on the 2016 WHO Classification of Tumors of the CNS (2016 WHO) [14].

### Genetic analysis

Tumor DNA was extracted using a DNeasy Blood & Tissue Kit (Qiagen, Tokyo, Japan). Details of genetic analysis, including PCR and sequencing for each gene status, were previously reported [19]. The presence of hotspot mutations in *IDH1* (R132) and *IDH2* (R172) was assessed by Sanger sequencing in all cases [19]. The two mutation hotspots in the *TERT* promoter were also analyzed by Sanger sequencing [12]. The copy number status of 1p–19q was determined by multiplex ligation-dependent probe amplification (MLPA) (Oligodendroglioma 1p–19q probemix and EK1 reagent kit, MRC-Holland, Amsterdam, Netherlands). The methylation status of the *MGMT* promoter was analyzed by quantitative methylation specific PCR (qMSP) after bisulfite modification of genomic DNA [20]. Based on an outcome-based study to determine an optimal cutoff to judge *MGMT* promoter methylation in a series of newly diagnosed glioblastomas (GB), we used a cut-off of ≥ 1% for *MGMT* methylation.

### Statistical analysis

Statistical analysis was performed using an SAS package and JMP Pro version 12 (SAS Institute, Cary, NC, USA). Categorized data were compared between subgroups using Chi square test. Overall survival curves were estimated by Kaplan–Meier method and compared with log-rank test. Univariate and multivariate analyses of risk factors were performed using Cox proportional hazards model. A P-value < 0.05 was considered statistically significant.

## Results

### Clinical characteristics

Table 1 shows clinical characteristics of the140 patients analyzed in this study. There were 78 males (55.7%) and 62 females (44.3%) with a median age of 75 years (range 70–93 years). Based on integrated diagnosis of 2016 WHO Classification, 114 patients (81.4%) were grade IV, 19 (13.6%) were III, and 7 (5.0%) were II [14]. Preoperative KPS scores ranged from 30 to 100 (median 70), and 67 patients (47.9%) had a score of ≥ 80. Regarding EOR, 28 patients (20.0%), 20 (14.3%), 55 (39.3%), and 37 (26.4%) underwent GTR, STR, PR, and biopsy, respectively. After resection, 96 patients (68.6%), 24 (17.1%), and 8 (5.7%) received combined RT and TMZ (RT + TMZ), TMZ monotherapy and RT alone, respectively.

**Table 1:** Clinical and molecular characteristics of the cohort

Characteristic (n = 140)	Data	(%)
Age (years)		
Median (range)	75 (70–93)	
70–74	62	(44.3)
75–79	41	(29.3)
80–	37	(26.4)
Gender		
Male	78	(55.7)
Female	62	(44.3)
WHO grade		
Grade II	7	(5.0)
Grade III	19	(13.6)
Grade IV	114	(81.4)
Preoperative KPS score		
80–100	67	(47.9)
–70	72	(51.4)
Unknown	1	(0.7)
Extent of surgical resection		
GTR	28	(20.0)
STR	20	(14.3)
PR	55	(39.3)
Biopsy	37	(26.4)
Adjuvant treatment		
RT + TMZ	96	(68.6)
TMZ monotherapy	24	(17.1)
RT alone	8	(5.7)
None	12	(8.6)
Radiation dose (Gy)		
50–60	78	(75.0)
< 50	20	(19.2)
Unknown	6	(5.8)
Genetic status		
*MGMT* promoter		
Methylated	68	(48.6)
Unmethylated	72	(51.4)
*IDH1/2*		
Wild type	129	(92.1)
Mutant	11	(7.7)
*TERT* promoter mutation	84 / 138	(60.9)
1p/19q codeletion	7 / 59	(11.9)
*TP53* mutation	50 / 140	(35.7)
2016 WHO classification		
*Diffuse astrocytoma, IDH-wildtype*	4	
Anaplastic astrocytoma, IDH-mutant	1	
*Anaplastic astrocytoma, IDH-wildtype*	12	
Glioblastoma, IDH-wildtype	112	
Gliosarcoma	1	
Glioblastoma, IDH-mutant	1	
Oligodendroglioma, IDH-mutant and 1p/19q-codeleted	3	
Anaplastic oligodendroglioma, IDH-mutant and 1p/19q-codeleted	4	
*Anaplastic oligodendroglioma, NOS* (lacking IDH-mutation and 1p/19q-codeletion)	2	

*WHO* World Health Organization, *KPS* Karnofsky Performance Status

Sixty-six patients (47.1%) were admitted within 2 years from the time of analysis (Online Resources 1, 2). Observation period ranged from 0.7 to 48.5 months (median 10.6 months). Tumor progression was observed in 79 patients (56.4%). 67 patients (47.9%) were deceased at the time of analysis. Median progression-free survival (mPFS) was 8.4 months (8.2 months in grade IV), and median overall survival (mOS) was 14.7 months (13.6 months in grade IV).

Table 2 shows treatment regimen according to age, preoperative KPS score and WHO grade. Distribution of EOR, especially tumor reduction versus biopsy, was not significantly different among groups in age, preoperative KPS score and WHO grade. Regardless of higher age (≥ 80 years) and lower KPS score (< 70 years), maximum and safe resection was intended to perform. Adjuvant treatment regimen was not significantly associated with preoperative KPS score but with both age and WHO grade. Patients (≥ 80 years) were significantly less likely to receive radiation (p < 0.0001).

**Table 2:** Treatment regimen according to age, preoperative KPS score and WHO grade

	Age				Preoperative KPS score		
	70–74	75–79	80–	p-value	80–100	–70	p-value
Total number	62	41	37		69	72	
Extent of surgical resection				0.50			0.51
GTR	9 (14.5%)	11 (26.8%)	8 (21.6%)		17 (25.4%)	11 (15.3%)	
STR	12 (19.4%)	4 (9.8%)	4 (10.8%)		9 (13.4%)	11 (15.3%)	
PR	27 (43.6%)	15 (36.6%)	13 (35.1%)		24 (35.8%)	31 (43.1%)	
Biopsy	14 (22.6%)	11 (26.8%)	12 (32.4%)		17 (25.4%)	19 (26.4%)	
Adjuvant treatment				< 0.0001*			0.80
RT + TMZ	56 (90.3%)	28 (68.3%)	12 (32.4%)		49 (73.1%)	47 (65.3%)	
TMZ monotherapy	4 (6.5%)	5 (12.2%)	15 (40.5%)		10 (14.9%)	14 (19.4%)	
RT alone	1 (1.6%)	5 (12.2%)	2 (5.4%)		3 (4.5%)	4(5.6%)	
None	1 (1.6%)	3 (7.3%)	8 (21.6%)		5 (7.5%)	7 (9.2%)	

	WHO grade			
	Grade II	Grade III	Grade IV	p-value
Total number	7	19	114	
Extent of surgical resection				0.08
GTR	3 (42.9%)	3 (15.8%)	22 (19.3%)	
STR	0 (0%)	2 (10.5%)	18 (15.8%)	
PR	2 (28.6%)	4 (21.1%)	49 (43.9%)	
Biopsy	2 (28.6%)	10 (52.5%)	25 (21.9%)	
Adjuvant treatment				0.0004*
RT + TMZ	2 (28.6%)	11 (57.9%)	83 (72.8%)	
TMZ monotherapy	1 (14.3%)	4 (21.1%)	19 (16.7%)	
RT alone	0 (0%)	2 (10.5%)	6 (5.3%)	
None	4 (57.1%)	2 (10.5%)	6 (5.3%)	

*Pearson’s Chi square test was applied for the statistical analysis. p < 0.05, significant difference

### Molecular characteristics

Table 1 also shows molecular characteristics and frequencies of each genetic status. *MGMT* promoter methylation was present in 68 tumors (48.6%). *IDH1*/*2* mutation was detected in only 11 tumors (7.7%). Notably, *IDH1*/*2* was wild-type in 4 of 7 grade II gliomas (57.1%) and 13 of 18 grade III (72.2%) (Online Resource 3). 1p/19q co-deletion was seen in 7 of 59 tumors (11.9%). *TERT* promoter mutations were observed in 84 of 138 tumors (60.9%), including 7 oligodendroglial tumors (7/8, 87.5%) and 69 GB (69/113, 61.1%) (Online Resource 3).

Results of integrated diagnosis according to the 2016 WHO Classification are shown in Table 1. Almost all grade IV gliomas were GB, *IDH*-wildtype (98.2%). Grade II and III astrocytomas consisted of 16 *IDH*-wildtype (94.1%) and 1 *IDH*-mutant (5.9%). In nine oligodendroglial tumors, two anaplastic oligodendroglioma (AO) lacked both *IDH* mutation and 1p/19q codeletion.

Arita et al. proposed molecular classification based on *IDH* and *TERT* mutation, which divided into four molecular groups, each showing distinct patient characteristics, histology, or clinical outcome [12]. The combined *IDH*/*TERT* classification is shown in Online Resource 3. There were a small number in *IDH* mutant (IDHmut) groups (10/138, 7.2%) regardless of *TERT* mutation status. The group with mutations in both *IDH* and *TERT* consisted of only oligodendroglial tumors (6/6, 100%). The group with mutation in *IDH* but not *TERT* contained two anaplastic astrocytoma and two GB. In *IDH* wild-type (IDHwt) groups, there were a larger number of tumors harboring *TERT* mutation (75/128, 58.6%) than those without mutation. In these groups, GB was the most common (45/53, 84.9% and 66/75, 88%).

### Treatment outcomes and prognostic factors

**Fig. 1:**
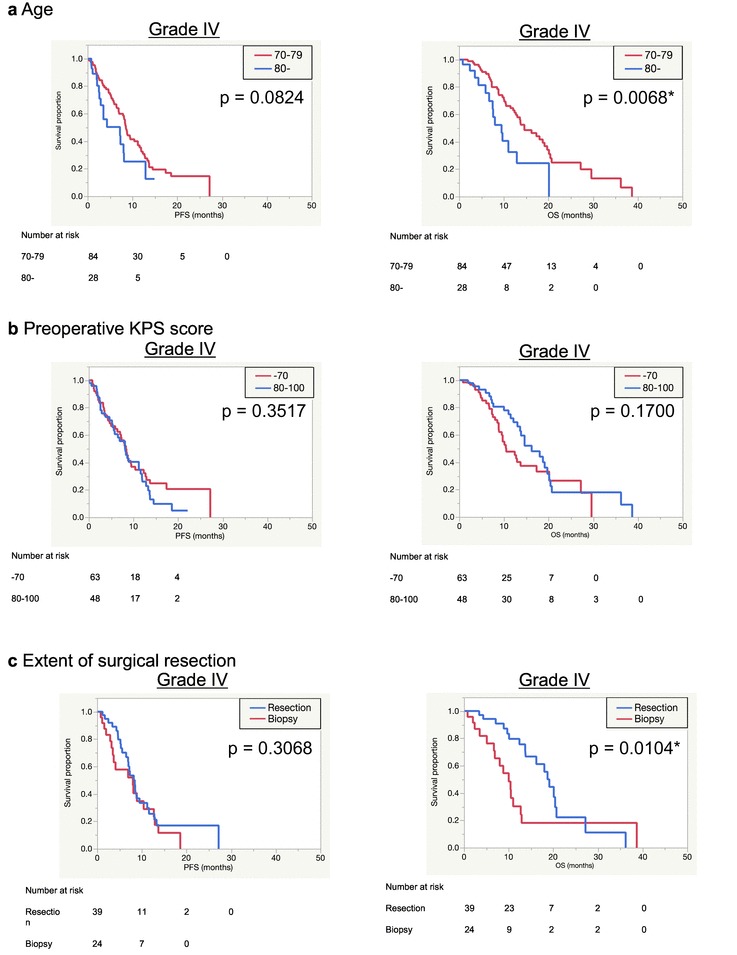

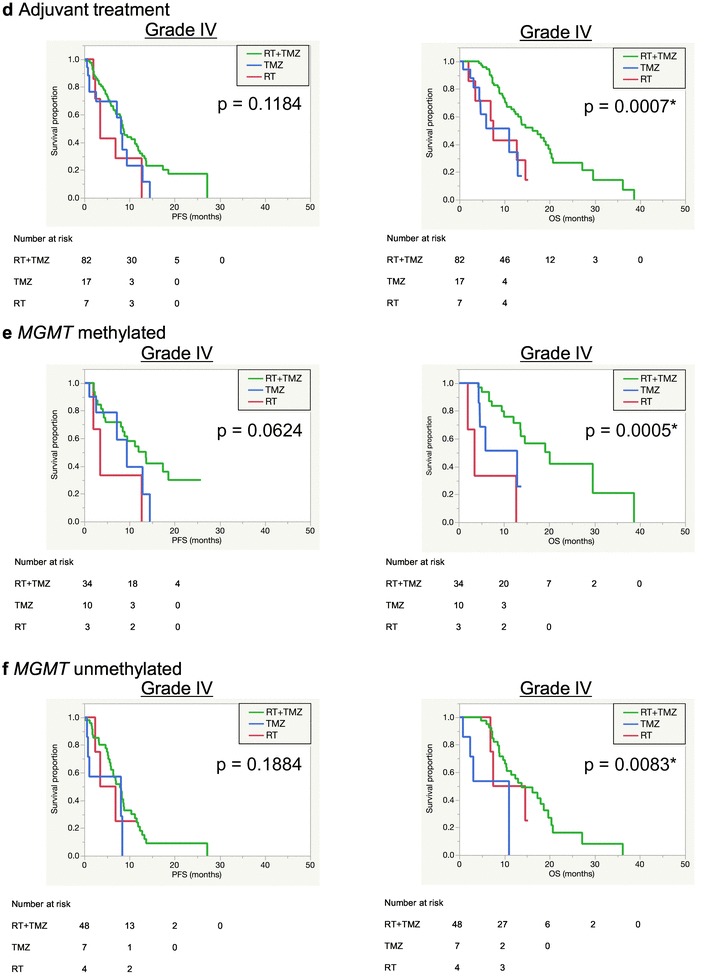

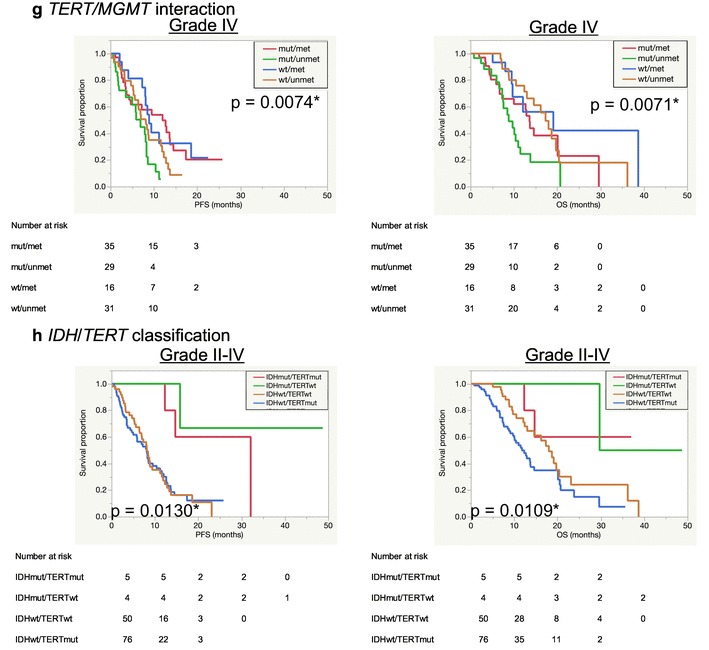
Kaplan–Meier survival curves according to age (**a**), preoperative KPS score (**b**), extent of surgical removal (**c**) and adjuvant treatment (**d**), adjuvant treatments in *MGMT* promotor methylated (**e**) or unmethylated (**f**) patients, *TERT*/*MGMT* interaction (**g**) in WHO Grade IV cases and *IDH*/TERT classification in WHO Grade II-IV cases (**h**)

Table 3 and Fig. 1 show median survival times and Kaplan–Meier survival curves of WHO grade IV cases, respectively. Table 3 also includes the results of univariate analyses of the relationships between factors and PFS/OS. Patients (≥ 80 years) had significantly shorter OS (9.5 months) compared to 14.6 months in those < 80 years old (p = 0.0068) (Fig. 1a and Online Resource 4a). Patients with preoperative KPS score of < 70 tended to survive for a shorter time than those with KPS 80–100 (10.5 versus 16.2 months, p = 0.1700) (Fig. 1b). The group that underwent aggressive surgical resection (GTR + STR) had significantly longer OS (19.1 months) than those who received biopsy (10 months) (p = 0.0104) (Fig. 1c). Notably, the resection group had longer OS than those who received non-aggressive treatment (PR + biopsy) (13 months) and this difference was also significant (p = 0.0310) (Online Resource 4b).

**Table 3:** Results of univariate analyses of the relationship between factors and survivals in WHO Grade IV cases

Factors	Median progression-free survival times (months)	p-value	Median overall survival times (months)	p-value
WHO grade IV			13.6	
Age		0.0824		0.0068*
70–79	8.5		14.6	
80–	7.2		9.5	
Preoperative KPS score		0.3517		0.1700
80–100	8.2		16.2	
0–70	8.3		10.5	
Extent of surgical resection		0.3068		0.0104*
Resection	8.4		19.1	
Biopsy	8		10	
Adjuvant treatment		0.1184		0.0007*
RT + TMZ	8.6		16.2	
TMZ monotherapy	8.1		11	
RT alone	3.5		7.5	
Radiation dose		0.2878		0.3837
50–	8.6		16.2	
–50	6.5		13.8	
*MGMT* promoter		0.0129*		0.3240
Methylated	9.5		13.7	
Unmethylated	7		13	
*TERT* promoter		0.6869		0.0109*
Wild type	8.4		18	
Mutated	7.9		10.4	

*< 0.05, significant difference

Patients who received adjuvant RT + TMZ had mOS of 16.2 months, while mOS of those who received TMZ monotherapy or RT alone was 11 or 7.5 months. RT + TMZ group had significantly longer OS than others (p = 0.0007) (Fig. 1d).

*MGMT* promoter methylated group showed better PFS compared to unmethylated group (9.5 months compared to 7 months) (p = 0.0129), whereas no significant difference was found in OS between them (13.7 months vs. 10 months) (p = 0.3240) (Online Resource 5a). Between these groups, there was no significant difference in distribution of treatment regimen (Online Resource 6). Based on *MGMT* methylation status, estimated survival times according to adjuvant treatments were compared (Fig. 1e, f). RT + TMZ group had a significantly longer OS time than other groups in both *MGMT* methylated patients (20.1 months) (p = 0.0005) and in unmethylated patients (13.8 months) (p = 0.0083). Although the number of RT group (n = 5, mOS = 11.1 months) was too small for statistical analyses, significant difference was found between RT + TMZ and RT groups in *MGMT* unmethylated patients. TMZ group had a significantly shorter OS time than RT + TMZ group even in methylated groups (n = 14, mOS = 12.9 months).

Regarding *TERT* mutation status, there was a significant difference between wild-type and mutated groups (Table 3 and Online Resource 5b). TERTmut group had significantly shorter OS (10.4 months) than TERTwt group (18 months) (p = 0.0109). Association between *TERT* mutation and *MGMT* methylation was investigated (Online Resource 6 and Fig. 1g). As the result, the interaction between *TERT* and *MGMT* was significant (PFS, p = 0.0074; OS, p = 0.0071) and TERTmut/MGMTunmet group had the poorest prognosis (8.8 months), followed by TERTmut/MGMTmet group (13.6 months).

In the WHO grade IV cohort, there was only one case with *IDH* mutation. Despite the small number even in WHO grade II–IV cases, IDHmut group had significantly longer survival (mPFS = 27.2 months, mOS = 29.7 months) than IDHwt group (mPFS = 8.2 months, mOS = 13.8 months) (PFS, p = 0.0003; OS, p = 0.0175) (Online Resource 5c). Based on the *IDH*/*TERT* classification, Kaplan–Meier survival curves of the four groups are shown in Fig. 1h. There appeared to be four distinct curves (p = 0.0109), but the observation period was too short for conclusive statistical results in the IDHmut groups. Among the IDHwt groups, there was a significant difference in OS between TERTwt and TERTmut groups (p = 0.0316) and IDHwt/TERTmut group had the shortest OS (11.8 months).

For WHO grade II–IV cases, univariate and multivariate analyses identified WHO grade as an independent prognostic factor (Online Resources 7, 8). As the results of univariate analysis of the relationships between characteristics and estimated survival times for WHO grade IV cases, age, resection, adjuvant treatment and *TERT* mutation status were significantly associated with longer OS (Table 3; Fig. 1 and Online Resource 4). Table 4 shows the results of multivariate analysis of factors associated with OS. Resection and adjuvant treatment (RT + TMZ) were identified as independent factors for good prognosis.

**Table 4:** Results of multivariate analyses of factors associated with overall survival in WHO Grade IV cases

Factors	Hazard ratio (95% Cl)	p-value
Age (< 80)	0.6128 (0.3776–1.815)	0.8161
Extent of surgical resection (resection)	0.5052 (0.2726–0.8903)	0.0175*
Adjuvant treatment (RT + TMZ)	0.4105 (0.2032–0.8701)	0.0209*
*TERT* promoter (wild-type)	0.5890 (0.3345–1.013)	0.0559

*p < 0.05, significant difference

## Discussion

In this study, we retrospectively analyzed 140 elderly patients with diffuse gliomas treated at 13 hospitals in the Kansai Network. The cohort had several clinical and molecular characteristics: age ≥ 80 years (26.4%), lower-grade glioma (18.6%), preoperative KPS scores below 70 (51.4%), resection (34.3%), adjuvant RT + TMZ (68.6%), *MGMT* promoter methylation (48.6%), *IDH1*/*2* mutation (7.7%) and *TERT* promoter mutation (60.9%). Higher age (≥ 80 years) and *TERT* promoter mutated were associated with poor prognosis. Resection and adjuvant RT + TMZ were identified as independent good prognostic factors.

Large cohort studies of elderly patients with diffuse gliomas are limited [4]. This study included 140 patients with an age of ≥ 70 years. Statistical analyses reconfirms previous reports that age is one of the most important prognostic factors and performance status is independently associated with survival [4, 21].

As the result according to 2016 WHO Classification, several characteristics were notable. First, grade II/III astrocytomas consisted of IDH-wt (94.1%) and IDH-mut (5.9%). Generally, the great majority falls into the IDH-mut category and IDH-wt astrocytomas are uncommon [22]. Moreover, in oligodendroglial tumors, some AO did not retain both *IDH* mutations and 1p/19q codeletion (22%). These tumors, histologically typical oligodendrogliomas, were diagnosed as AO, NOS after careful evaluation.

Arita et al. reported that almost all tumors harboring concurrent *IDH1*/*2* mutations and total 1p/19q loss had *TERT* promoter mutations [23]. Indeed, in *TERT* mutated tumors, all tumors with *IDH* mutation harbored 1p/19q codeletion. Moreover, they proposed combined *IDH*/*TERT* classification [12]. Also in this study, IDHwt/TERTmut patients expected the shortest survival in four subgroups.

Wiestler et al. reported that *MGMT* promoter methylation was 35% in malignant astrocytoma in the elderly (> 65 years) [24]. Our cohort had a relatively high frequency of *MGMT* methylation (48.6%). Grade IV patients with *MGMT* methylated tumors showed better survival compared to those with unmethylated tumors, but the difference did not reach statistical significance. This trend was also observed in previous reports [4, 8].

*TERT* mutation status had a prognostic impact in this study. Together with *MGMT* methylation status, significant interaction between *TERT* and *MGMT* was observed. GB patients with *TERT* mutated and *MGMT* unmethylated had the poorest prognosis. Based on the results, a combination of *IDH, TERT*, and *MGMT* would refine clinically relevant classification of elderly diffuse gliomas.

Several treatment options have been recommended [16]. For low-grade gliomas in the elderly, adjuvant treatments have never been discussed. In the cohort, non-aggressive resection tended to be undertaken. On the other hand, adjuvant RT + TMZ was conducted in the majority. Notably, there was no significant difference in survival time between 50 and 60 Gy and < 50 Gy RT groups, which is discussed in several studies [6, 8, 25, 26]. TMZ monotherapy group resulted in shorter survival regardless of *MGMT* status, as was inconsistent with other reports [17].

TMZ concomitant with and adjuvant to RT is a widely used approach, but the role in elderly cases remains discussed [27]. Arvold et al. stated that the addition of TMZ to RT was associated with a small survival gain [28]. Franceschi et al. reported that RT + TMZ is effective only in methylated *MGMT* tumors [29]. On the other hand, in the phase 3 trial by Perry et al., the addition of TMZ to short-course RT was associated with significantly longer survival [3]. In subgroup analyses, the benefit was also observed in unmethylated *MGMT* cases [3]. These results suggest that the addition of TMZ to RT confers a survival benefit regardless of *MGMT* status.

In general, treatment outcomes are mostly consistent with previous reports [4, 21, 30]. Although the optimal treatment remains controversial, maximum and safe resection followed by short course RT with concurrent and adjuvant TMZ is warranted in GB [3].

As a multi-institutional retrospective cohort design, there are several limitations. Unlike a randomized study, selection bias on decision-making of treatment strategy could exist. Attending physicians may decide to deliver treatments considering the patients’ age, conditions and wishes, and thus a selection could affect the survival findings. Variation of treatment regimen at multiple institutions, such as radiation protocol and dose schedule, should also be considered. The limited number of patients could explain the absence of statistical power to detect differences between groups. Modest prognostic impact of molecular characteristics might be partly due to the limited follow-up period of the population.

In conclusion, we report characteristics and outcomes of elderly patients with diffuse gliomas in the Kansai Network. This community-based study elucidated the present status of real-world practice. Further investigation in a larger population would contribute to our better understanding of the pathogenesis of glioma in the elderly.

## Electronic supplementary material

Below is the link to the electronic supplementary material.

Supplementary material 1 (XLSX 67 KB)

Supplementary material 2 (DOCX 78 KB)

Supplementary material 3 (PPT 1132 KB)
