# Supplementary material for: Characteristics and outcomes of elderly patients with diffuse gliomas: a multi-institutional cohort study by Kansai Molecular Diagnosis Network for CNS Tumors
Source: J Neurooncol. 2018 Aug 3;140(2):329–39. doi: 10.1007/s11060-018-2957-7 (PMC6244782; doi:10.1007/s11060-018-2957-7)
Supplement: Supplementary file 2 — Supplementary material 2 (DOCX 78 KB) [file 11060_2018_2957_MOESM2_ESM.docx]

**Online Resource 2** Distribution of the patient numbers by admission year

| Admission Year | 2007 | 2008 | 2009 | 2010 | 2011 | 2012 | 2013 | 2014 | 2015 | 2016 | Total |
| --- | --- | --- | --- | --- | --- | --- | --- | --- | --- | --- | --- |
| Grade II | 0 | 0 | 0 | 0 | 2 | 0 | 0 | 1 | 2 | 2 | 7 |
| Grade III | 0 | 0 | 0 | 0 | 0 | 3 | 7 | 3 | 3 | 3 | 19 |
| Grade IV | 3 | 3 | 4 | 4 | 2 | 7 | 16 | 19 | 36 | 20 | 114 |
| Total | 3 | 3 | 4 | 4 | 4 | 10 | 23 | 23 | 41 | 25 | 140 |

**Online Resource 3** Molecular status of *IDH1/2* and *TERT* in the WHO grade

| *IDH1/2* mutation | *TERT* promoter mutation | WHO grade | | | Total |
| --- | --- | --- | --- | --- | --- |
|  |  | Grade II | Grade III | Grade IV |  |
| + | + | 3 | 3 | 0 | 6 |
| + | - | 0 | 2 | 2 | 4 |
| - | - | 0 | 8 | 45 | 53 |
| - | + | 4 | 5 | 66 | 75 |
| Total number of analyzed samples | | 7 | 18 | 113 | 138 |

**Online Resource 6** Treatment regimen and TERT promoter mutation status according to *MGMT* promoter methylation status in WHO Grade IV cases

|  | *MGMT* promoter methylation status | | |
| --- | --- | --- | --- |
|  | Methylated | Unmethylated | p-value |
| WHO grade IV | 52 | 61 |  |
| Extent of surgical resection |  |  | 0.96 |
| Gross total resection | 13 (19.1%) | 15 (20.8%) |  |
| Subtotal resection | 9 (13.2%) | 11 (15.3%) |  |
| Partial resection | 28 (41.2%) | 27 (37.5%) |  |
| Biopsy | 18 (26.5%) | 19 (26.4%) |  |
| Adjuvant treatment |  |  | 0.14 |
| Radiation + Temozolomide | 42 (61.8%) | 54 (75.0%) |  |
| Temozolomide monotherapy | 14 (21.4%) | 10 (13.9%) |  |
| Radiation alone | 3 (4.4%) | 5 (6.9%) |  |
| None | 9 (13.2%) | 3 (4.2%) |  |
| *TERT* promoter mutation status |  |  | 0.03* |
| mutated | 36 | 30 |  |
| wild-type | 16 | 31 |  |

* Pearson’s chi-square test was was applied for the statistical analysis. p < 0.05, significant difference

**Online Resource 8** Results of univariate and multivariate analyses of factors associated with overall survival in WHO Grade II-IV cases

| Factors | Univariate | |  | Multivariate | |
| --- | --- | --- | --- | --- | --- |
|  | Hazard ratio (95%Cl) | p-value |  | Hazard ratio (95%Cl) | p-value |
| Age (< 80) | 0.4422 (0.2508-0.8251) | 0.0118* |  | 0.4799 (0.2435-0.9732) | 0.0420* |
| WHO grade  (Grade II/III) | 0.5507 (0.3177-0.9122) | 0.0197* |  | 0.3507 (0.1588-0.7018) | 0.0023* |
| Preoperative KPS score (80-100) | 0.6409 (0.4046-1.0194) | 0.0602 |  |  |  |
| Extent of surgical removal_1 (Resection) | 0.7342 (0.4389-1.2808) | 0.2666 |  |  |  |
| Extent of surgical removal_2 (GTR+STR) | 0.5222 (0.3056-0.8621) | 0.0106* |  | 0.4434 (0.2511-0.7565) | 0.0025* |
| Adjuvant treatment  (RT + TMZ) | 0.5741 (0.3475-0.9816) | 0.0429* |  | 0.3804 (0.2073-0.7111) | 0.0027* |
| *MGMT* promoter (Methylated) | 0.5698 (0.3588-0.8953) | 0.0146* |  | 0.4611 (0.2730-0.7710) | 0.0032* |
| *IDH*1/2 (mutated) | 0.1135 (0.1846-0.3692) | <0.0001* |  | 0.8272 (0.2365-2.2101) | 0.7280 |
| *TERT* promoter (wild-type) | 0.8064 (0.5065-1.2721) | 0.3560 |  |  |  |

* p < 0.05, significant difference
