# Supplementary material for: Characteristics and outcomes of elderly patients with diffuse gliomas: a multi-institutional cohort study by Kansai Molecular Diagnosis Network for CNS Tumors
Source: J Neurooncol. 2018 Aug 3;140(2):329–39. doi: 10.1007/s11060-018-2957-7 (PMC6244782; doi:10.1007/s11060-018-2957-7)
Supplement: Supplementary file 3 — Supplementary material 3 (PPT 1132 KB) [file 11060_2018_2957_MOESM3_ESM.ppt]

## Slide 1
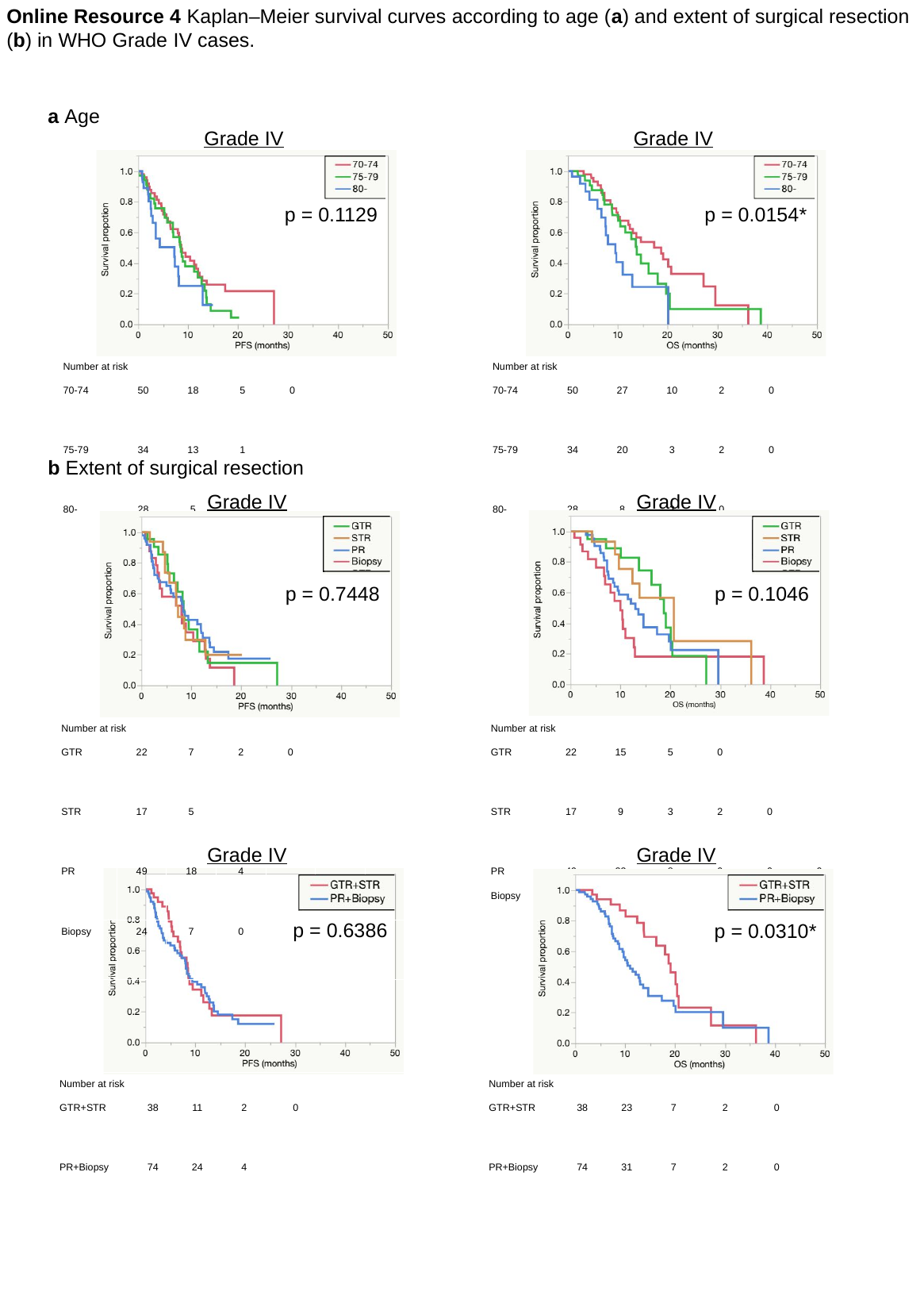

Online Resource 4 Kaplan–Meier survival curves according to age (a) and extent of surgical resection (b) in WHO Grade IV cases.
a Age
Grade IV
p = 0.1129
Grade IV
p = 0.0154*
| Number at risk | | | | | | |
| --- | --- | --- | --- | --- | --- | --- |
| 70-74 | 50 | 18 | 5 | 0 | | |
| 75-79 | 34 | 13 | 1 | | | |
| 80- | 28 | 5 | | | | |
| Number at risk | | | | | | |
| --- | --- | --- | --- | --- | --- | --- |
| 70-74 | 50 | 27 | 10 | 2 | 0 | |
| 75-79 | 34 | 20 | 3 | 2 | 0 | |
| 80- | 28 | 8 | 2 | 0 | | |
b Extent of surgical resection
Grade IV
p = 0.7448
Grade IV
p = 0.1046
| Number at risk | | | | | | |
| --- | --- | --- | --- | --- | --- | --- |
| GTR | 22 | 7 | 2 | 0 | | |
| STR | 17 | 5 | | | | |
| PR | 49 | 18 | 4 | | | |
| Biopsy | 24 | 7 | 0 | | | |
| Number at risk | | | | | | |
| --- | --- | --- | --- | --- | --- | --- |
| GTR | 22 | 15 | 5 | 0 | | |
| STR | 17 | 9 | 3 | 2 | 0 | |
| PR | 49 | 23 | 8 | 0 | 0 | 0 |
| Biopsy | 24 | 9 | 2 | 2 | 0 | |
Grade IV
Grade IV
p = 0.0310*
p = 0.6386
| Number at risk | | | | | | |
| --- | --- | --- | --- | --- | --- | --- |
| GTR+STR | 38 | 11 | 2 | 0 | | |
| PR+Biopsy | 74 | 24 | 4 | | | |
| Number at risk | | | | | | |
| --- | --- | --- | --- | --- | --- | --- |
| GTR+STR | 38 | 23 | 7 | 2 | 0 | |
| PR+Biopsy | 74 | 31 | 7 | 2 | 0 | |

## Slide 2
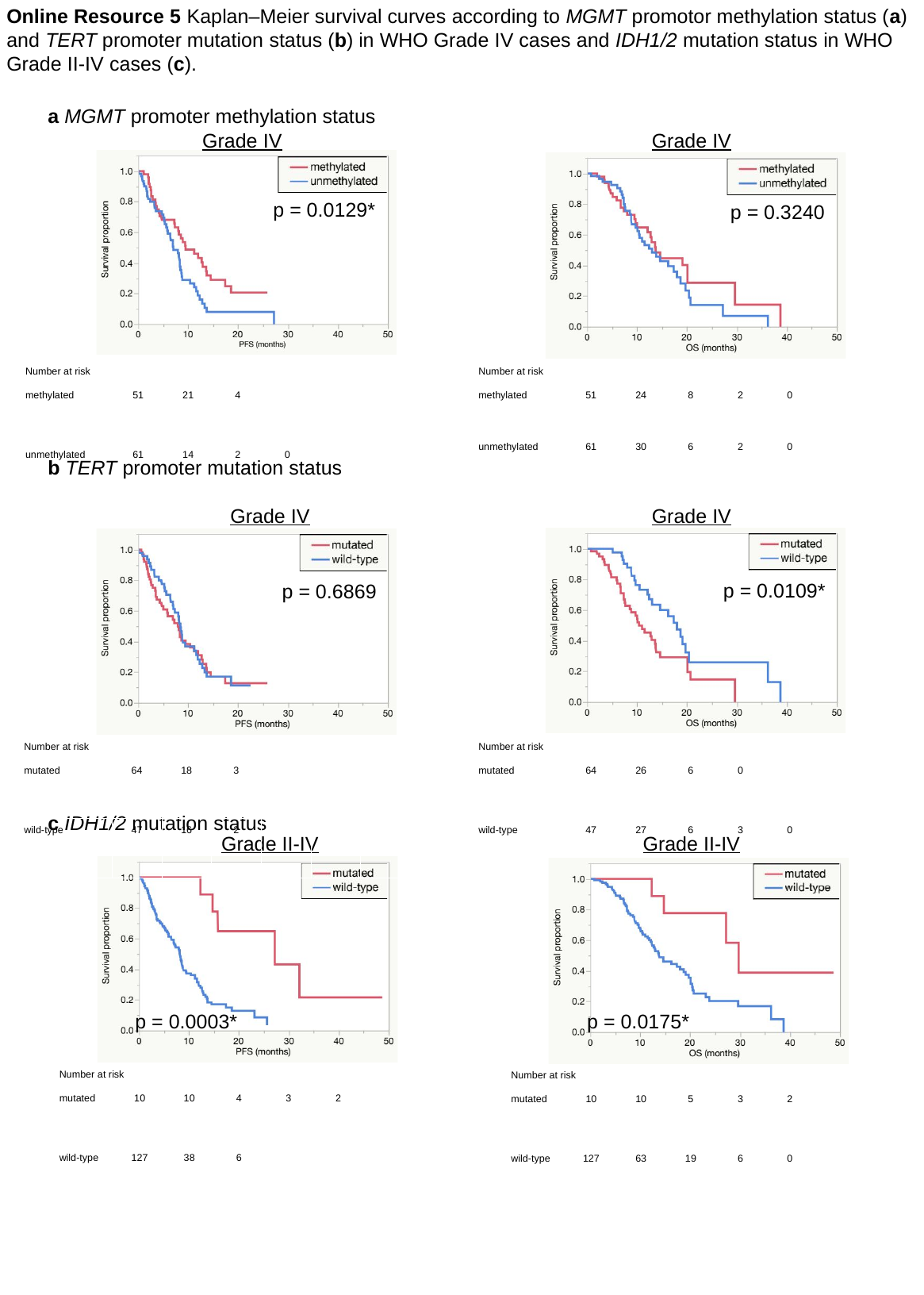

Online Resource 5 Kaplan–Meier survival curves according to MGMT promotor methylation status (a) and TERT promoter mutation status (b) in WHO Grade IV cases and IDH1/2 mutation status in WHO Grade II-IV cases (c).
a MGMT promoter methylation status
Grade IV
Grade IV
p = 0.0129*
p = 0.3240
| Number at risk | | | | | | |
| --- | --- | --- | --- | --- | --- | --- |
| methylated | 51 | 21 | 4 | | | |
| unmethylated | 61 | 14 | 2 | 0 | | |
| Number at risk | | | | | | |
| --- | --- | --- | --- | --- | --- | --- |
| methylated | 51 | 24 | 8 | 2 | 0 | |
| unmethylated | 61 | 30 | 6 | 2 | 0 | |
b TERT promoter mutation status
Grade IV
Grade IV
p = 0.0109*
p = 0.6869
| Number at risk | | | | | | |
| --- | --- | --- | --- | --- | --- | --- |
| mutated | 64 | 18 | 3 | | | |
| wild-type | 47 | 16 | 2 | | | |
| Number at risk | | | | | | |
| --- | --- | --- | --- | --- | --- | --- |
| mutated | 64 | 26 | 6 | 0 | | |
| wild-type | 47 | 27 | 6 | 3 | 0 | |
c IDH1/2 mutation status
Grade II-IV
Grade II-IV
p = 0.0003*
p = 0.0175*
| Number at risk | | | | | | |
| --- | --- | --- | --- | --- | --- | --- |
| mutated | 10 | 10 | 4 | 3 | 2 | |
| wild-type | 127 | 38 | 6 | | | |
| Number at risk | | | | | | |
| --- | --- | --- | --- | --- | --- | --- |
| mutated | 10 | 10 | 5 | 3 | 2 | |
| wild-type | 127 | 63 | 19 | 6 | 0 | |

## Slide 3
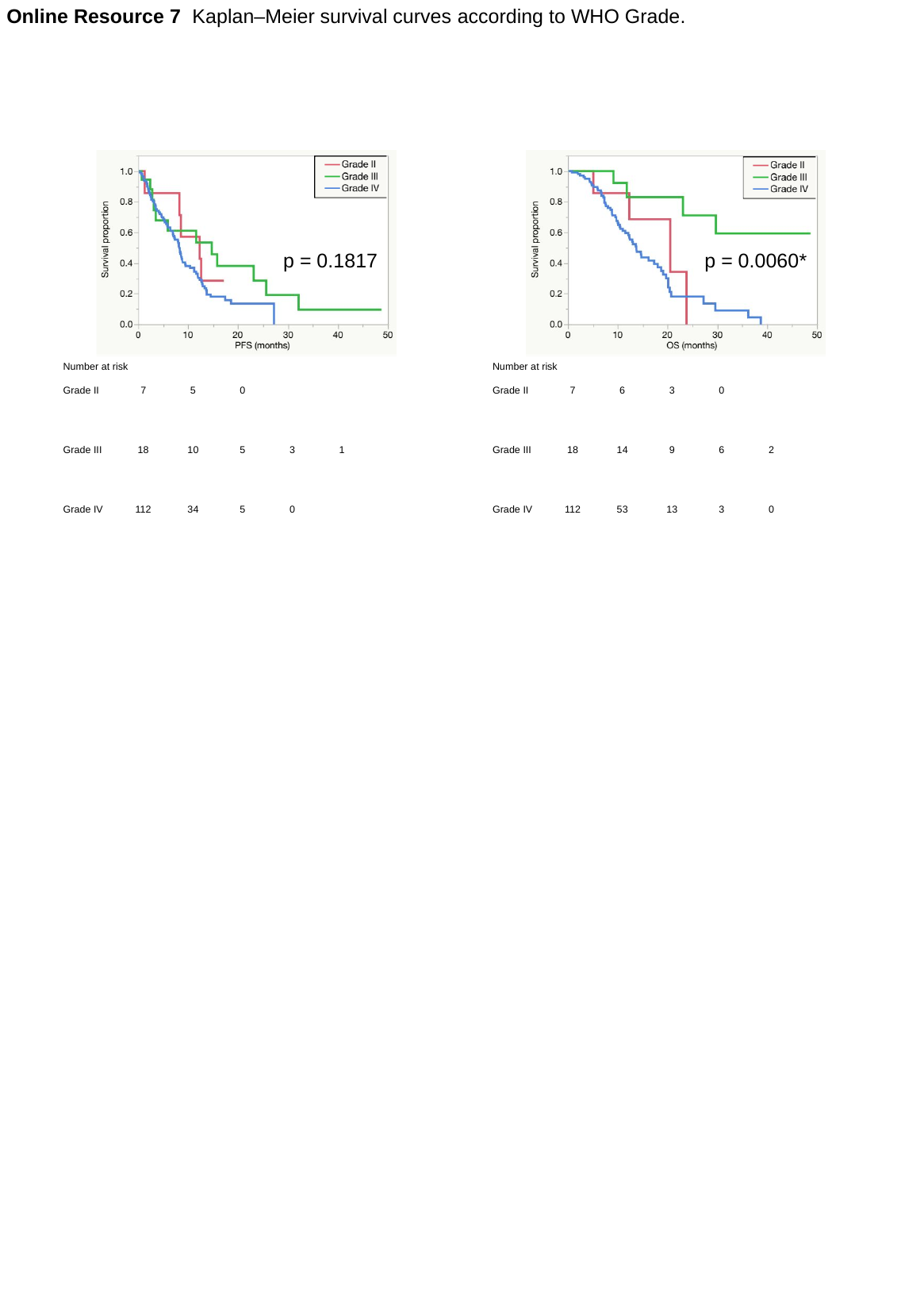

Online Resource 7 Kaplan–Meier survival curves according to WHO Grade.
p = 0.1817
p = 0.0060*
| Number at risk | | | | | | |
| --- | --- | --- | --- | --- | --- | --- |
| Grade II | 7 | 5 | 0 | | | |
| Grade III | 18 | 10 | 5 | 3 | 1 | |
| Grade IV | 112 | 34 | 5 | 0 | | |
| Number at risk | | | | | | |
| --- | --- | --- | --- | --- | --- | --- |
| Grade II | 7 | 6 | 3 | 0 | | |
| Grade III | 18 | 14 | 9 | 6 | 2 | |
| Grade IV | 112 | 53 | 13 | 3 | 0 | |
